# Long-term consumption of caffeine-free high sucrose cola beverages aggravates the pathogenesis of EAE in mice

**DOI:** 10.1038/celldisc.2017.20

**Published:** 2017-06-20

**Authors:** Guangchao Cao, Qian Wang, Wanjun Huang, Jiyu Tong, Dewei Ye, Yan He, Zonghua Liu, Xin Tang, Hao Cheng, Qiong Wen, Dehai Li, Hau-Tak Chau, Yiming Wen, Hui Zhong, Ziyu Meng, Hui Liu, Zhenzhou Wu, Liqing Zhao, Richard A Flavell, Hongwei Zhou, Aimin Xu, Hengwen Yang, Zhinan Yin

**Affiliations:** 1 The First Affiliated Hospital, Biomedical Translational Research Institute, Guangdong Province Key Laboratory of Molecular Immunology and Antibody Engineering, Jinan University, Guangzhou, China; 2 Joint Institute of Metabolic Medicine between State Key Laboratory of Pharmaceutical Biotechnology, The University of Hong Kong and Jinan University, Guangzhou, China; 3 State Key Laboratory of Pharmaceutical Biotechnology, The University of Hong Kong, Hong Kong, China; 4 State Key Laboratory of Organ Failure Research, Division of Laboratory Medicine, Zhujiang Hospital, Department of Environmental Health, School of Public Health, Southern Medical University, Guangzhou, China; 5 State Key Laboratory of Biotherapy, Collaborative Innovation Center for Biotherapy, West China Hospital, Sichuan University, Chengdu, China; 6 State Key Laboratory of Medicinal Chemical Biology, College of Life Sciences, Nankai University, Tianjin, China; 7 Department of Immunobiology, School of Medicine, Yale University, New Haven, USA

**Keywords:** high sucrose, cola beverages, microbiota, Th17, EAE

## Abstract

Epidemiological data provide strong evidence of dramatically increasing incidences of many autoimmune diseases in the past few decades, mainly in western and westernized countries. Recent studies clearly revealed that ‘Western diet’ increases the risk of autoimmune diseases at least partially via disrupting intestinal tight junctions and altering the construction and metabolites of microbiota. However, the role of high sucrose cola beverages (HSCBs), which are one of the main sources of added sugar in the western diet, is barely known. Recently, a population study showed that regular consumption of sugar-sweetened beverages is associated with increased risk of seropositive rheumatoid arthritis in women, which provokes interest in the genuine effects of these beverages on the pathogenesis of autoimmune diseases and the underlying mechanisms. Here we showed that long-term consumption of caffeine-free HSCBs aggravated the pathogenesis of experimental autoimmune encephalomyelitis in mice in a microbiota-dependent manner. Further investigation revealed that HSCBs altered community structure of microbiota and increased Th17 cells. High sucrose consumption had similar detrimental effects while caffeine contamination limited the infiltrated pathogenic immune cells and counteracted these effects. These results uncovered a deleterious role of decaffeinated HSCBs in aggravating the pathogenesis of experimental autoimmune encephalomyelitis in mice.

## Introduction

Autoimmune diseases such as multiple sclerosis (MS), rheumatoid arthritis (RA), type I diabetes, inflammatory bowel disease and psoriasis are multifactorial etiological diseases, involving T cell-mediated inflammatory pathology. Genetic factors are closely correlated with the development of autoimmune diseases [1, 2], but this could not explain the dramatically increasing incidences of these diseases in the past few decades, mainly in western and westernized countries [3]. Besides, studies in monozygotic twins showed a relatively low-concordance rate for most of the diseases [4], suggesting that environmental factors also play important roles in the pathogenesis of autoimmune diseases.

Recently, emerging works on the role of the gastrointestinal (GI) microbiota in the pathogenesis of autoimmune diseases were reported, providing clear evidence that dysbiosis of the microbiota is associated with these diseases [5,6,7, 8,9,10,11]. Depleting microbiota aborted the pathogenesis of experimental autoimmune encephalomyelitis (EAE) [12, 13], the most widely used animal model for human MS, suggesting a critical role of microbiota in EAE disease. Further investigations demonstrated that gut-residing segmented filamentous bacterium antigens, presented by DC, specifically drive Th17 cells differentiation, which is pivotal for the pathogenesis of EAE and RA [14,15,16,17]. Moreover, intestinal luminal adenosine 5′-triphosphate (ATP), which could be derived from commensal bacteria, was shown to activate a unique subset of lamina propria cells, CD70^high^ CD11c^low^ cells, leading to the differentiation of Th17 cells and an increasing risk for inflammatory colitis [18] and EAE [19]. These results corroborated GI microbiota and metabolites as pivotal internal environmental elements for triggering autoimmune diseases.

Dietary patterns have short- and long-term effects in shaping the composition of gut microbiota and modulating its metabolic machinery products. Increasing evidence suggests that dietary modulation of the microbiome is involved in the pathogenesis of autoimmune diseases such as inflammatory bowel disease and osteomyelitis [20,21,22,23]. The ‘Western diet’, characterized as high-fat and cholesterol, high-protein, high-sugar and excess salt intake, as well as frequent consumption of processed and ‘fast foods’ with industrial food additives, was found to increase the risk of many of these autoimmune diseases [21,23,24,25,26,27,28,29,30]. Among these dietary factors, high fat and industrial emulsifiers have been found to trigger the disease at least partially via impacts on microtioba [23, 31,32,33,34]. Recently, consumption of sugar-sweetened soda (including high sugar contained regular cola, caffeine-free cola and other sugar-sweetened carbonated soda), but not diet soda (sugar-free), was reported to be associated with an increased risk of seropositive RA in women [35], suggesting a pro-inflammatory effect of refined sugars in pathogenesis. In fact, sugar consumption has rapidly increased in lower-middle and upper-middle-income countries in Asia, and carbonated soft drinks are the most significant vectors for the increasing sugar consumption [36]. However, we still lack the knowledge regarding the genuine effects of sugary cola beverages and the refined sugars in the pathogenesis of autoimmune diseases and whether the consumption of these beverages has any impact on the construction of GI microbiota.

Here we adopted a mouse model of EAE to assess the effects of several most popular HSCBs (regular Coca-Cola, caffeine-free Coca-Cola (Coca-Free), regular Pepsi, and caffeine-free Pepsi (Pepsi-Free)) and diet soda (Coca-Zero) on the pathogenesis of autoimmune diseases. The ingredients of these beverages are listed in Table 1. Results showed that long-term consumption of caffeine-free cola beverages (Coca-Free and Pepsi-Free), but not other cola beverages, aggravated disease development, as indicated by higher clinical scores, exacerbated demyelization and elevated central nervous system (CNS)-infiltrated pathogenic Th17 cells. Further research revealed that all the HSCBs and high sucrose markedly changed the community structure of intestinal microbiota to promote Th17 cells and feces transplantation from these groups also increased the risk of EAE. Caffeine, on the other hand, inhibited the number of CNS-infiltrated lymphocytes and counteracted the detrimental effects of high sucrose on both active- and passive-induced EAE. Moreover, we also found elevated luminal ATP levels in mice consuming HSCBs or high sucrose, but not Coca-zero. Taken together, the aforementioned findings converged to strongly support a detrimental potential of high sucrose in HSCBs on the pathogenesis of EAE through modulating the construction of intestinal microbiota, while caffeine contamination in regular HSCBs counteracts this effect and is protective.

## Results

### Long-term consumption of caffeine-free HSCBs aggravated the pathogenesis of EAE

The introduction of HSCBs has dramatically changed the dietary component throughout the world during the last century. In an attempt to study the effects of long-term consumption of these beverages on autoimmune diseases, we built on an observation using the EAE model. We chose several of the most popular commercial HSCBs with similar concentration of sucrose (regular Coca-Cola, regular Pepsi, caffeine-free Coca-Cola (Coca-Free) and caffeine-free Pepsi (Pepsi-Free)) and none-sucrose cola (Coca-Zero) for study. The list of ingredients of these beverages is shown in Table 1.

Mice were given individual cola beverages in the dark phase to drink freely for 8 weeks and then the metabolic parameters were monitored using metabolic cages. Results showed that mice were in fond of these cola beverages as indicated by liquid intake (Supplementary Figure S1a). The metabolic status was also altered as shown by chow intake, respiratory exchange ratio, spontaneous locomotor activity and energy expenditure (Supplementary Figure S1b–j). However, 8-week consumption of individual cola beverages did not alter the body weight gain when compared with water controls (Supplementary Figure S1k), which suggested a metabolic balance in these mice.

**Figure 1:**
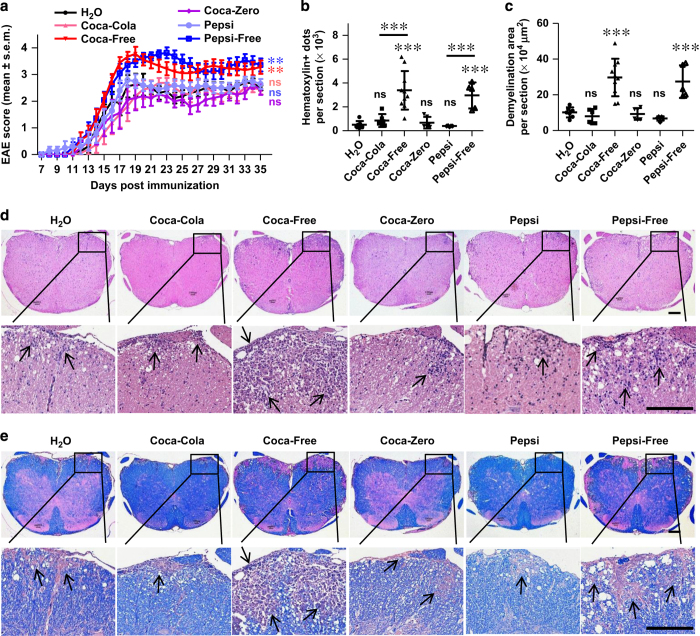
Long-term consumption of caffeine-free HSCBs aggravates the pathogenesis of EAE. (**a**) Mice were treated with individual cola beverages for 8 weeks and immunized with MOG_35-55_ for the induction of EAE. Clinical score was assessed daily and shown (*n*=10). (**b**–**e**) 18 days post immunization, lumbosacral spinal cords were isolated and performed H&E (**d**) or Luxol fast blue (**e**) staining for assessment of histopathology. Representative sections (**d**, **e**) and statistical analysis (**b**, **c**) data are shown. Infiltration of lymphocytes and demyelination are highlighted by arrow, and scale bars are present as 250 μm. All data above are representative of three independent experiments. ns, not significant.

We next induced EAE in mice after treatment with individual cola beverages for 8 weeks. Results showed that long-term consumption of Coca-Free or Pepsi-Free significantly exacerbated the disease compared with water control, as evidenced by elevated disease score (Figure 1a), increased lymphocyte infiltration (Figure 1b and d) and more severe demyelination (Figure 1c and e). However, the disease activity in response to regular Coca-Cola, regular Pepsi or Coca-Zero was not significantly changed (Figure 1a–e). We also induced EAE in mice receiving limited amount of these beverages (5 ml per mouse per day). Results showed that limited amount of Coca-Free and Pepsi-Free still aggravated the disease in the early stage (Supplementary Figure S2). These results indicated that long-term consumption of caffeine-free HSCBs increased the risk for EAE disease in mice.

### Pathogenic role of Th17 cells in the progress of aggravated EAE in response to caffeine-free HSCBs

**Figure 2:**
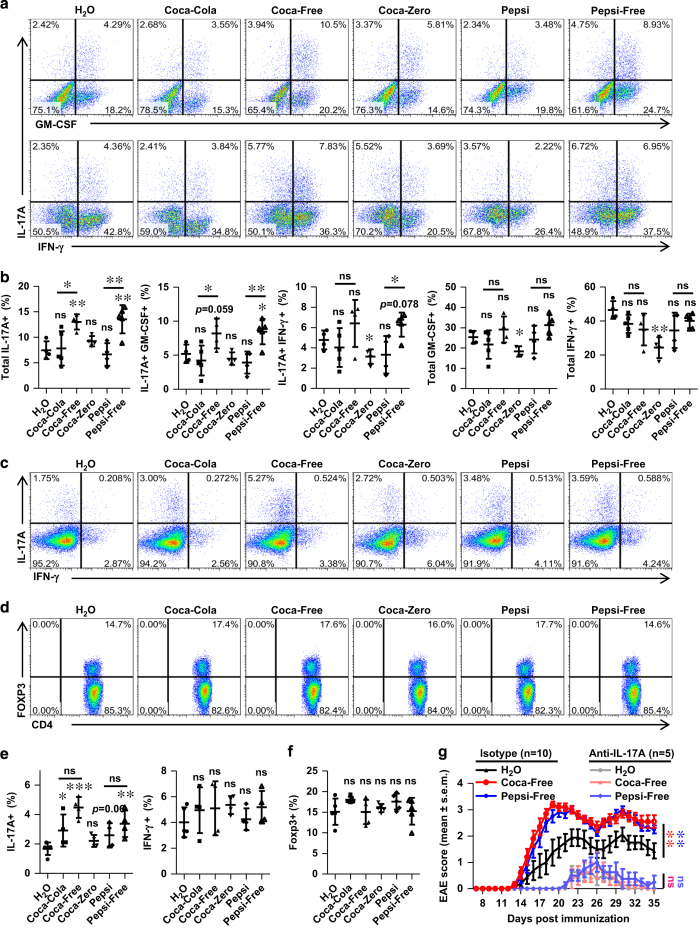
HSCBs boost Th17 responses in EAE. Lymphocytes from CNS or inguinal LN were isolated 18 days post immunization and used for assessment of different CD4 T cell subsets. (**a**) Representative staining of CNS-infiltrated inflammatory CD4 T cell subsets, gated on TCRβ^+^ CD4^+^. (**b**) Statistical analysis of the percentages of IL-17A^+^ GMCSF^+^ double positive, IL-17A^+^ IFN-γ^+^ double positive, total IL-17A^+^ (both IL-17A^+^ GMCSF^−^ and IL-17A^+^ GMCSF^+^), total GMSCF^+^ (both IL-17A^−^ GMCSF^+^ and IL-17A^+^ GMCSF^+^) and total IFN-γ^+^ (both IL-17A^−^ IFN-γ^+^ and IL-17A^+^ IFN-γ^+^) in **a**. (**c** and **d**) Representative staining of different CD4 T cell subsets in inguinal LN, gated on TCRβ^+^ CD4^+^. (**e** and **f**) Statistical analysis of data in **c**, **d**. (**g**) Mice consuming individual beverages were i.p. injected with anti-IL-17A antibodies (*n*=5) or isotype control antibodies (*n*=10) in the induction and duration of EAE. The clinical score was assessed daily and shown. All data above are representative of at least two independent experiments.

Th17 cells are critical for the pathogenesis of EAE via producing pro-inflammatory cytokines, such as IL-17A, GM-CSF and IFN-γ, which directly induce pathogenesis or recruit neutrophils and macrophages and lead to myelin damage [37, 38]. To investigate whether caffeine-free HSCBs consumption aggravates the pathogenesis of EAE by modulating these cells, lymphocytes from the CNS and inguinal lymph nodes (LN) were isolated and the levels of Th17 cells were detected. As suspected, mice consuming Coca-Free and Pepsi-Free displayed elevated CNS-infiltrating pro-inflammatory Th17 cells (IL-17A^+^ GMCSF^+^ double positive, IL-17A^+^ IFN-γ^+^ double positive or total IL-17A^+^ (both IL-17A^+^ GMCSF^−^ single positive and IL-17A^+^ GMCSF^+^ double positive), Figure 2a and b), while the levels of Th17 cells in other groups were either unchanged (Coca-Cola and Pepsi) or even slightly decreased (Coca-Zero for IL-17A^+^ IFN-γ^+^). The CNS-infiltrating total GM-CSF^+^ (both IL-17A^−^ GMCSF^+^ single positive and IL-17A^+^ GMSCF^+^ double positive) or total IFN-γ^+^ (both IL-17A^−^ IFN-γ^+^ single positive and IL-17A^+^ IFN-γ^+^ double positive) CD4 T cells were also calculated, which were unaffected in all HSCB groups but decreased in mice consuming Coca-Zero (Figure 2a and b). The Th17 level in the LN of Coca-Free and Pepsi-Free groups was also elevated, but still unchanged in response to Coca-Zero, which was consistent with CNS results (Figure 2c and e). Surprisingly, the numbers of Th17 cells in LN were also elevated in response to regular Coca-Cola and regular Pepsi (Figure 2c and e), suggesting a detrimental potential of these beverages. The IFN-γ^+^ percentage in CD4 T cells was also unaffected in the LN of HSCB groups (Figure 2c and e), which was consistent with CNS results.

We also detected the percentage of Treg cells in the LN, which were reported to suppress the pathogenesis of EAE via inhibiting the function of Th17 cells [39]. No significant difference of Treg cells was detected among all these groups (Figure 2d and f). These results suggested that HSCBs specifically enhanced Th17 response without affecting the percentage of Treg cells.

To confirm the pathogenic role of IL-17A in this process, we blocked the effector function of IL-17A via neutralizing antibodies in the initiation and duration of EAE. Blocking IL-17A significantly ameliorated the pathogenesis of EAE and abrogated the exacerbation of disease activity in response to Coca-Free or Pepsi-Free (Figure 2g). Collectively, these results demonstrated a pathogenic role of Th17 cells in the detrimental effects of decaffeinated HSCBs in EAE.

### HSCBs alters the community structure of intestinal microbiota

**Figure 3:**
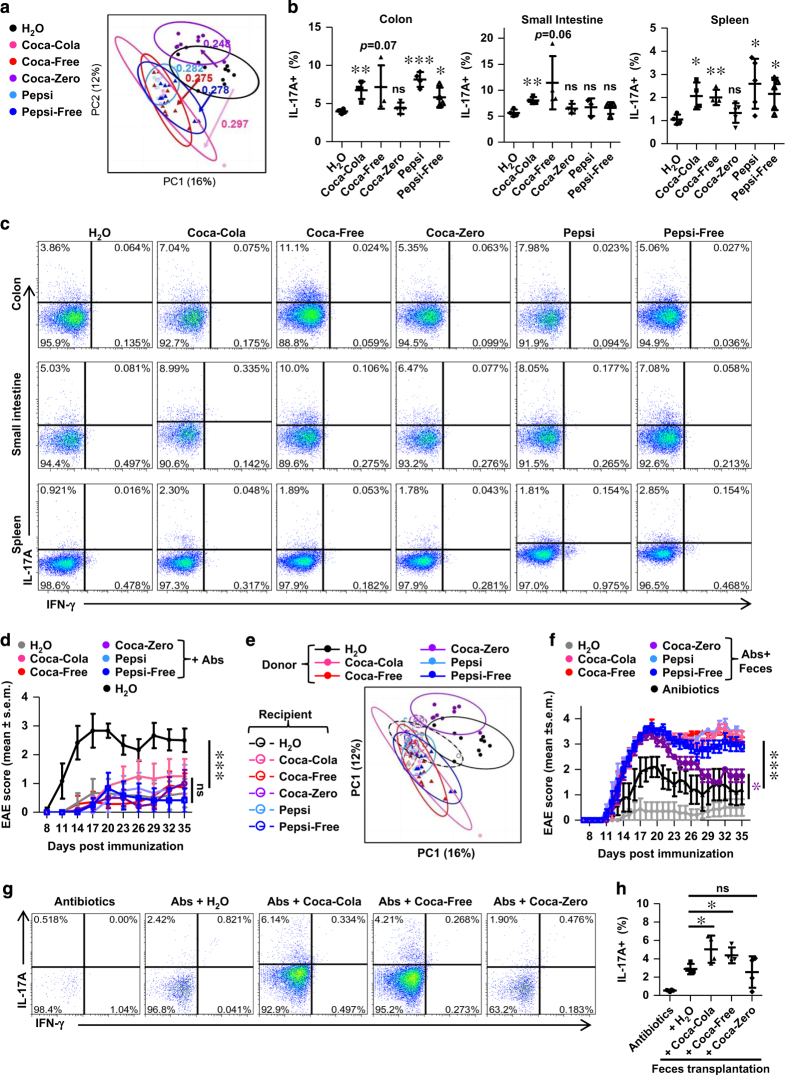
HSCBs induce a disease-prone structure of microbiota. (**a**) Mice were treated with individual cola beverages for 8 weeks and feces were used for isolation of 16s rRNA and subsequent sequencing analysis. Unweighted Unifrac principal coordinates analysis plots of each sample and the distance of each group are shown (*n*=10). Statistical analysis (**b**) and representative staining (**c**) of IL-17 in CD4 T cells isolated from different organs of mice without immunization, gated on TCRβ^+^ CD4^+^. (**d**) Mice were depleted of microbiota after Cola beverages consumption and immunized for EAE disease. Clinical score was assessed and shown (*n*=7). (**e**) Microbiota-depleted mice were transplanted with feces from mice consuming individual cola beverages or water and 16s rRNA analysis of the recipient mice was performed. (**f**) The feces recipient mice in (**e**) were induced for EAE disease. Clinical score was assessed and shown (*n*=7). (**g** and **h**) Representative staining and statistical analysis of Th17 level in the colon in response to fecal transplantation, gated on TCRβ^+^ CD4^+^. All data above are representative of at least two independent experiments. Abs, antibiotics.

Intestinal microbiota have been shown to profoundly affect the development of Th17 and pathogenesis of EAE, and dietary pattern was reported to alter these intestinal commensals due to their plasticity. To address whether cola beverages alter the community structure of microbiota, we performed 16S rRNA sequencing analysis of feces DNA isolated from mice consuming these beverages or H_2_O for 8 weeks. On the basis of unweighted Unifrac beta diversity analysis, all cola beverages significantly altered the community structure of intestinal microbiota, and all HSCBs groups showed similar shift patterns (Figure 3a, Supplementary Figure S3a and Supplementary Table S1). In addition, HSCBs consumption led to numerous enriched taxa, such as genus Escherichia, Mucispirillum, Butyricicoccus, Coprococcus, Desulfovibrio, Odoribacter, Pareprevotella and Ruminococcus (Supplementary Figure S3a). Several other taxa were decreased after HSCBs consumption (Supplementary Table S1). The shift patterns of commensals in response to Coca-Zero have both similarities and discrepancies when compared with HSCBs. Moreover, we discovered the species *Escherichia coli, Mucispirillum schaedleri, Butyricicoccus pullicaecorum, Desulfovibrio C21_c20* and *Ruminococcus gnavus* were also enriched in HSCBs-consuming mice (Supplementary Table S1). These data revealed that HSCBs consumption had selective effects on specific microbial taxa.

Among the enriched commensals in response to HSCBs, *Escherichia coli* was reported to instigate chronic colitis and Th17 response [40,41,42,43], Ruminococcus was shown to drive provocative IL-17 expression in mesenteric lymph nodes [44], and Mucispirillum elicited T cells-dependent IgA response, similar to segmented filamentous bacterium [45].

Consistent with altered microbiota community structure, the Th17 response in the intestine was also increased in mice consuming HSCBs (Figure 3b and c). Besides, the Th17 level in the spleen was also increased in these mice before EAE induction (Figure 3b and c), which was dovetailed with elevated Th17 response in the LN of EAE mice (Figure 2c and e). The Th17 level was not significantly changed in either intestine or spleen of mice consuming Coca-Zero before EAE induction (Figure 3b and c), which was also consistent with the LN results of EAE mice (Figure 2c and e). Collectively, these results further strengthen a detrimental potential of all HSCBs but not Coca-Zero in provoking Th17 response.

To determine the role of intestinal flora in the hypersensitivity of EAE in response to caffeine-free HSCBs, mice were depleted of microbiota via antibiotics before the induction of EAE. Consistent with previous studies [12, 13], mice were much less susceptible to EAE after clearance of microbiota. Besides, none of the cola beverages rendered mice more vulnerable to EAE in the absence of intestinal flora (Figure 3d), suggesting that microbiota were required for mice to be hypersensitive to EAE after HSCBs consumption.

To further address whether the alterations of intestinal commensals correlate with aggravated pathogenesis of EAE, we harvested the feces from mice consuming individual cola beverages and transferred into antibiotics-pre-treated recipients. 16s rRNA sequencing analysis was performed from donors at 8 and 11 weeks of cola beverages consumption to confirm the consistency of micriobiota structure (Supplementary Figure S3c). 16s rRNA sequencing analysis of intestinal microbiota from recipients was also performed after 3-week continuous transfer. Results showed that the shift pattern of microbiota structure in the recipients was similar as the donor, but the alteration was less dramatic (Figure 3e, Supplementary Figure S3b and Supplementary Table S2). More importantly, the susceptibility to EAE could be transferred via feces transplantation in all HSCBs feces recipients, which confirmed the pathogenic commensal structure in response to all HSCBs (Figure 3f). This was further evidenced by increased Th17 response in both regular Coca-Cola and Coca-Free feces recipients (Figure 3g and h). We also found slightly increased susceptibility to EAE in recipients of feces from Coca-Zero group, even though the Th17 level was unchanged, which suggested a different mechanism.

Collectively, these results strongly demonstrated a tight link of alternated microbiota in response to HSCBs with exacerbated pathogenesis of EAE.

### High-sucrose consumption aggravated pathogenesis of EAE

**Figure 4:**
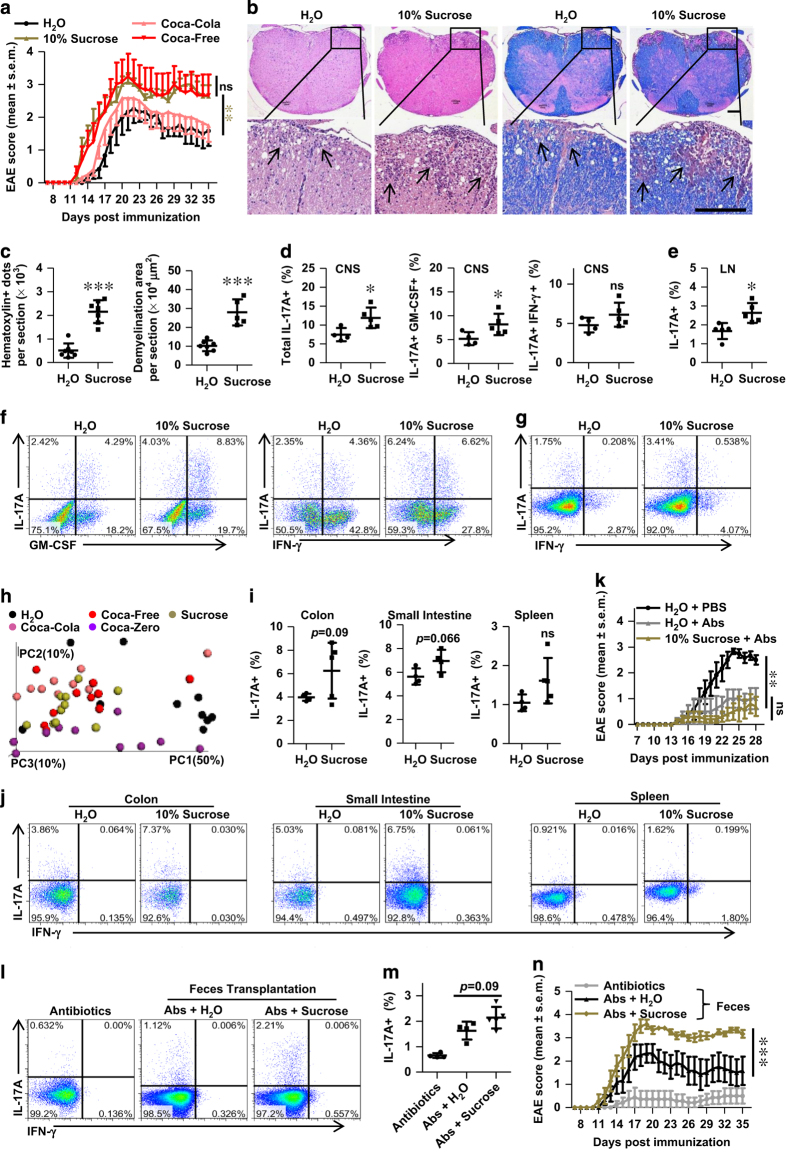
Long-term consumption of high sucrose exacerbates EAE disease. (**a**) Clinical score of mice consuming 10% sucrose (w/v), different HSCBs or H_2_O control (*n*=10) after immunizaiton. (**b** and **c**) Representative histological sections of spin cords isolated from mice on day 18 after immunization and quantitative analysis of disease severity. Infiltration of lymphocytes and demyelination are highlighted by arrow, and scale bars are present as 250 μm. (**d**–**g**) Representative staining and statistical analysis of Th17 cells in the CNS (**d** and **f**) and inguinal LN (**e** and **g**) on day 18 post immunization. Represented dots were gated on TCRβ^+^ CD4^+^. (**h**) Three-dimensional Unweighted Unifrac principal coordinates analysis plots of fecal samples isolated from mice consuming individual beverages for 8 weeks (*n*=10). (**i** and **j**) Representative staining and statistical analysis of Th17 cells in different organs of mice consuming 10% sucrose or H_2_O. Represented dots were gated on TCRβ^+^ CD4^+^. (**k**) Clinical EAE score of mice with clearance of microbiota before immunization (*n*=7). (**l** and **m**) Microbiota depleted mice were transferred with feces from mice consuming 10% sucrose or H_2_O, the Th17 level in the colon was assessed and shown. (**n**) Feces recipients as in (**l** and **m**) were also immunized for EAE, and the clinical score was assessed and shown (*n*=7). All data above are representative of at least two independent experiments.

All HSCBs studied above contained high level of sucrose (10–11% w/v), and the shift patterns of microbiota and IL-17 in response to these HSCBs were similar, but different with Coca-Zero, which was sucrose-free. Besides, consumption of sugar-sweetened soda, but not diet soda, was reported to be associated with an increased risk of seropositive RA in women [35]. Thus we studied the effects of pure high sucrose (10% w/v) in these processes. Metabolic studies of mice consuming high sucrose mimicked the changes in HSCBs groups (Supplementary Figure S4). More importantly, mice were also more susceptible to EAE in response to 10% sucrose (Figure 4a–c). But there was no significant difference between Coca-Free and 10% sucrose groups (Figure 4a), suggesting that high sucrose is the main detrimental component in these beverages. Besides, the Th17 response was also enhanced in both CNS and inguinal LN (Figure 4d–g).

16s rRNA sequencing analysis also revealed a similar shift pattern of microbiota structure in the feces of mice consuming 10% sucrose compared with HSCBs and the species *Mucispirillum schaedleri, Butyricicoccus pullicaecorum, Desulfovibrio C21_c20* and *Ruminococcus gnavus* were also enriched (Figure 4h, Supplementary Figure S5a and Supplementary Table S1). This was further evidenced with a trend to increase the Th17 cells in intestine in response to high sucrose (Figure 4i and j). Clearance of microtioba also eliminated the detrimental role of high sucrose (Figure 4k) and fecal transplantation transferred the susceptibility to EAE in recipients of high sucrose feces. The luminal Th17 level was also slightly increased in response to high sucrose feces (Figure 4l–n), which matched with our hypothesis.

Sucrose was digested in the small intestine and dissected to fructose and glucose, and glucose was reported to trigger ATP secretion from bacteria [46]. Luminal ATP was shown to promote Th17 cells differentiation in lamina propria and promote the pathogenesis of colitis and EAE [18, 19]. Thus we also detected the luminal ATP level after long-term consumption of individual cola beverages or 10% sucrose. Results showed that luminal ATP was significantly increased in response to HSCBs and 10% sucrose, but not Coca-zero (Supplementary Figures S3d and 5b). Depleting microbiota almost erased all luminal ATP and eliminated the elevation in response to HSCBs or 10% sucrose (Supplementary Figures S3e and 5c). These results indicated that high sucrose increased luminal ATP in a microbiota-dependent way. Indeed, fecal transplantation restored luminal ATP in antibiotics-treated recipients. However, fecal transplantation from mice consuming HSCBs or high sucrose did not boost luminal ATP when compared with H_2_O controls (Supplementary Figures S3f and 5d). These results suggested that high sucrose might directly stimulate the release of ATP in a microtiota-dependent manner rather than through enriching ATP-producing taxa. These results also suggested that the detrimental role of altered microbiota in response to HSCBs or high sucrose was not limited by ATP level. Whether elevated ATP plays any role in the aggravated pathogenesis of EAE needs further investigation.

Collectively, these results demonstrated that high sucrose in HSCBs altered the community structure of microbiota, increased Th17 responses and potentially increased the risk of EAE.

### Caffeine contamination counteracted the detrimental role of sucrose

**Figure 5:**
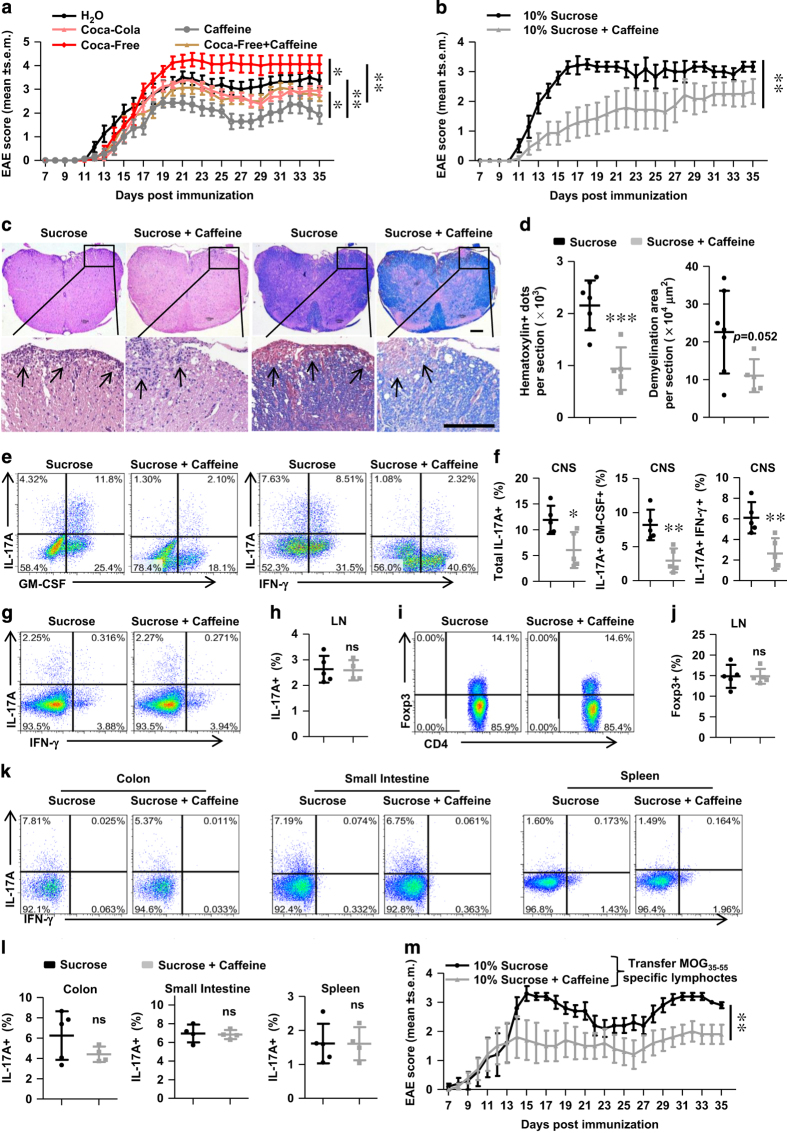
Caffeine counteracts the disease-prone potential of high sucrose in EAE. (**a**) Coca-Free was replenished with caffeine (0.1 mg ml^−1^) and used for treatment of mice for 8 weeks before immunization. The clinical score was assessed and shown. *n*=8. (**b**) Caffeine (0.1 mg ml^−1^) was added to the 10% sucrose solution and used for treatment of mice for 8 weeks before immunization. The clinical score was assessed and shown. *n*=7. (**c** and **d**) Representative histological sections and quantitative analysis of disease severity of mice as in **b**. Infiltration of lymphocytes and demyelination are highlighted by arrow, and scale bars are present as 250 μm. (**e** and **f**) The Th17 level in the CNS was assessed on day 18 post immunization, a representative staining and statistical analysis are shown. Cells were gated on TCRβ^+^ CD4^+^. (**g**–**j**) The Th17 and Treg levels were also detected in the inguinal LN on day 18 post immunization. Cells were gated on TCRβ^+^ CD4^+^. A representative staining and statistical analysis are shown. (**k** and **l**) Representative staining and statistical analysis of Th17 cells in different organs of mice under disease-free condition are shown. Cells were gated on TCRβ^+^ CD4^+^. (**m**) Mice consuming 10% sucrose or caffeinated 10% sucrose were transferred with MOG_35-55_ specific lymphocytes (2×10^6^ cells per mice) for passive-induced EAE. The clinical score was assessed and shown. *n*=5. All data above are representative of at least two independent experiments.

While all HSCBs altered the community structure of microbiota and enhanced peripheral Th17 responses, only caffeine-free HSCBs exacerbated the pathogenesis of EAE. Several researchers have reported a protective role of caffeine in delaying and reducing the onset and severity of EAE via inhibiting the infiltration of inflammatory lymphocytes [47,48,49]. This raised the hypothesis that caffeine contamination in regular Coca-Cola and regular Pepsi might ameliorate the pathogenesis of EAE and counteract the effects of high sucrose. Thus, we replenished caffeine (0.1 mg ml^−1^) in Coca-Free and tested its effect on the pathogenesis of EAE. Results showed that caffeine consumption reduced the severity of EAE, which was consistent with previous reports [ 47,48,49]. Besides, replenishing caffeine in Coca-Free ameliorated the severity of disease to a level similar as regular Coca-Cola (Figure 5a), but the disease in these two groups were still more severe than the caffeine (in H_2_O) group, which further strengthened the detrimental potential of these beverages.

To further investigate the effects of caffeine on the detrimental role of high sucrose, we added caffeine (0.1 mg ml^−1^) to 10% sucrose and used for treatment in parallel with 10% sucrose. As suspected, caffeine addition delayed the onset and reduced the severity of EAE (Figure 5b–d). Indeed, the CNS-infiltrated inflammatory lymphocytes were significantly reduced in response to caffeine contamination (Figure 5e and f), consistent with previous findings in regular Coca-Cola and regular Pepsi (Figures 1d, 2a and b). However, the Th17 level was unaffected in the inguinal LN of EAE mice (Figure 5g and h), neither in the colon, small intestine or spleen of disease-free mice (Figure 5k and l). Caffeine addition did not alter the Treg level either (Figure 5i and j). Collectively, these results suggested that caffeine counteracted the detrimental role of sucrose via reducing infiltrated inflammatory lymphocytes without altering peripheral Th17 responses. This was further evidenced by ameliorated pathogenesis in response to caffeine contamination in a passive-induced EAE model via transferring MOG_35-55_ specific lymphocytes (Figure 5m).

## Discussion

Multicellular organisms are comprised of both the macroscopic host and its symbiotic commensal microbiota. In humans, symbionts outnumber host cells by at least a factor of 10 and express at least 10-fold more unique genes than their host genome. These highly diverse and evolving microbes have intimate interactions with the host immune system, and this immune system-microbiota alliance allows the induction of protective responses to pathogens and continuous tolerance to innocuous antigens. However, overuse of antibiotics and dramatic changes in diet over the last century may have greatly disturbed the homeostasis of this symbiotic relationship, synchronizing with markedly increasing incidences of many human diseases, including autoimmune and inflammatory disorders.

HSCB is one of the leading food sources of added sugar intakes in the western diet [50] and is the most prevalent vector for increased sugar consumption in Asia [36]. Our current research uncovered that long-term consumption of caffeine-free HSCBs exacerbated the pathogenesis of EAE (Figures 1 and 2), a classic mouse model of autoimmune diseases. Interrogation of microbiota composition with 16S rRNA sequencing and use of the unweighted Unifrac algorithm to compare community structure revealed that intestinal flora were disturbed after prolonged consumption of HSCBs. Additionally, this disturbance was pathogenic, as revealed by feces transplantation experiments (Figure 3). Moreover, high sucrose played an important role in the detrimental effects of HSCBs (Figure 4), while caffeine contamination counteracted these effects via limiting CNS-infiltrated lymphocytes (Figure 5).

In our report, HSCBs consumption augmented numerous organisms (Supplementary Table S1) and many of these alterations were reproducible in response to high sucrose (Supplementary Table S1) or upon fecal transplantation (Supplementary Table S2). Several IL-17-provocating bacteria such as *Escherichia coli* and Ruminococcus were enriched in response to HSCBs [ 40,41,42,43,44]. Mucispirillum, which elicited T cells-dependent IgA response, was also among the enriched taxa [45]. These data revealed that HSCBs had selective effects on specific microbial taxa with similarities to high sucrose, and these alterations might correlate with aggravated pathogenesis of autoimmune diseases. However, direct evidence linking the aforementioned bacterial taxa with the pathogenesis of EAE or human MS is still lacking. Meta analysis of the GI microbiota constitution of MS patients and colonization of individual-correlated organisms in germ-free mice can lead to more specific phenotypic outcomes and experimental observations that will help to elucidate their genuine effects on the pathogenesis of MS and EAE. Besides, microbiota might also undergo metabolic alterations after HSCBs consumption and metagenomics analysis of these organisms will better illustrate this issue.

The community structure of microbiota was also significantly altered upon diet beverage (Coca-Zero) consumption, with both similarities and discrepancies compared with HSCBs (Supplementary Table S1). Besides, recipients were also slightly increased the susceptibility to EAE in response to Coca-Zero feces, but the Th17 response was not significantly altered (Figure 3f–h). These data suggested that Coca-Zero also had a detrimental potential, but might through a different mechanism. Coca-Zero lacks of sucrose but is supplied with artificial sweeteners, including sucralose, aspartame and acesulfame (analog of saccharin). Recently, metabolic abnormalities were found in mice that consumed artificial sweeteners such as sucralose, aspartame and saccharin via induction of compositional and functional alterations of the intestinal microbiota [51]. Whether these artificial sweeteners are involved in the detrimental potential effects of Coca-Zero needs further investigation. Besides, other ingredients of cola beverages may also have influence on microbiota. Unfortunately, due to the ‘patented’ formula of these commercial beverages, we were unable to address the effects of all these components.

Luminal ATP triggers Th17-prone molecules, such as IL-6, IL-23p19 and TGFβ-activating integrin-αV and -β8 from a CD70^high^ CD11c^low^ subset of the lamina propria cells, and administration of ATP exacerbates a T-cell-mediated colitis model with enhanced Th17 differentiation [18]. Luminal ATP was also linked with the pathogenesis of EAE [18, 19]. Digested in the small intestine, sucrose is dissected into fructose and glucose. Increased luminal ATP upon HSCBs or high sucrose but not Coca-Zero intake (Supplementary Figures S3d and 5b) matched with previous findings that high glucose triggered ATP secretion from multiple bacteria [46]. However, the elevation of ATP level was not detected in fecal transplanted recipients (Supplementary Figures S3f and 5d). These results suggested that the promotion of luminal ATP in response to HSCBs or high sucrose might not be due to the enriching ATP-producing taxa, but rather via a direct stimulation from microbiota. These results also suggested that the detrimental role of altered microbiota in mice consuming HSCBs or high sucrose was not limited by the ATP level (Figure 3e). Whether the augmented ATP plays any role in the exacerbated pathogenesis of EAE needs further investigation. The extracellular ATP receptors (that is, P2X and P2Y receptors) are highly expressed in numerous immune cells in various organs and tissues [52] and ATP could also directly induce excite-toxicity in oligodendrocytes via P2X7 receptors [53]. Thus, specific blocking of ATP receptors in the intestine or erasing luminal ATP will better address the role of ATP in this process.

The metabolic activities of mice consuming individual cola beverages or sucrose were also significantly altered (Supplementary Figures S1 and 4), and metabolic factors also regulated the development and function of T cells [54]. Whether the metabolic changes in response to HSCBs or high sucrose contributed to the detrimental role of these beverages needs further investigation.

In conclusion, our results revealed a disease-prone effect of caffeine-free HSCBs via alternating microbiota, which led to increased Th17 cells and aggravated pathogenesis of EAE. Contamination of caffeine in regular HSCBs counteracted the detrimental role of high sucrose via limiting infiltrated lymphocytes. Thus, our findings provide strong evidence that dietary modulation of microbiota is an etiological factor in the pathogenesis of autoimmune diseases and HSCBs, especially caffeine-free HSCBs are detrimental in this process.

## Materials and Methods

### Mice

C57BL/6J WT mice were purchased from Guangdong Medical Laboratory Animal Center (Guangzhou, China). All mice were maintained under specific pathogen free conditions at Jinan University (Guangzhou, China). All experiments were performed using female mice at 6–8 weeks old and in accordance with guidelines for animal care, created by Jinan University Experimental Animal Ethics Committee.

### Reagents

FITC- or PE-Cy5-conjugated anti-mouse CD4 (clone GK1.5), PE-conjugated anti-mouse IFN-γ (clone XMG1.2), FITC-, PE- or APC-conjugated anti-mouse IL-17A (clone 17F3), PE-conjugated anti-mouse GM-CSF (clone MP1-22E9), PE-Cy5-conjugated anti-mouse TCRβ (clone H57-597) were purchased from Sungene Biotech (Tianjin, China); PE-conjugated anti-mouse Foxp3 (clone MF-14) were purchased from Biolegend (San Diego, CA, USA); the myelin oligodendrocyte glycoprotein (MOG) 35–55 was purchased from GL Biochem Corporation (Shanghai, China); non-viable, desiccated Mycobacterium tuberculosis H37 RA was purchased from BD Difco (Detroit, MI, USA); pertussis toxin was purchased from List Biological Lab (Campbell, CA, USA); incomplete Freund adjuvant (IFA), Phorbol 12-myristate 13-acetate, Ionomycin, metronidazole, ampicillin, vancomycin hydrochloride and neomycin sulfate were purchased from Sigma Aldrich Company (St. Louis, MO); Amphotericin B was purchased from Solarbio Company (Beijing, China); ATP assay kit was purchased from Beyotime Biotech (Haimen, China).

### Cola beverages consumption

Mice were given individual cola beverages in the dark phase (0800–2 000) and water in the light phase (0800–2 000) to drink freely. Cola beverages consumption was performed 8 weeks before EAE induction and throughout the duration of disease progression. Body weight was recorded weekly.

### Metabolic cages

After consumption of different beverages for 8 weeks, mice were individually housed in metabolic chambers at 20–22 °C on a 12-h light/12-h dark cycle with lights on at 0800. Cola beverages were decarbonated and supplied in the dark phase same as mentioned above. Metabolic measurements (food intake, oxygen consumption and respiratory exchange rate) were recorded continuously using a comprehensive laboratory animal monitoring system (CLAMS, Columbus Instruments, Columbus, OH, USA). Liquid intake in the light phase was also recorded by CLAMS, while liquid intake in the dark phase was monitored manually. Spontaneous locomotor activity was monitored simultaneously and continuously over the measurement period by infrared beam sensors, separated by 1.27 cm from each other. Total caloric intake was summed as calories from chow (3.03 kcal g^−1^) and beverages (see Table 1). The energy expenditure was calculated with equation

$$\text{Energy}\;\text{expenditure}=\left(3.815+1.232\times \mathrm{RER}\right)\times {\mathrm{VO}}_{2}.$$

Data were collected over at least 3 days following at least 1 day of mice adaptation to the metabolic cages.

### Induction and assessment of EAE

Induction and assessment of EAE were performed as instructed [55, 56]. For active-inducing EAE, mice were subcutaneously immunized with 200 μg MOG_35-55_ peptide emulsified in CFA containing 5 mg ml^−1^ non-viable and desiccated *M. tuberculosis* H37 RA. Pertussis toxin (300 ng per mouse) in PBS was administered i.p. on day 0 and day 2. For passive-inducing EAE, MOG_35-55_ immunized mice were killed on day 10 and total lymphocytes from draining lymph nodes were isolated and cultured with MOG_35-55_ for 3 days. These MOG_35-55_ specific lymphocytes were isolated with Ficoll to remove dead cells and transferred into unimmunized mice (2×10^6^ cells per mice). Clinical assessment of EAE scores was performed daily on a scale of 0–5 as instructed [55].

### Histology

After 18 days post immunization, mice were fixed by heart perfusion with 4% (w/v) paraformaldehyde, and the lumbosacral spinal cords were obtained and embedded in paraffin. Sections (4 mm thick) that had been deparaffinized and rehydrated were stained with hematoxylin and eosin (H&E), or with Luxol fast blue (LFB) for analysis of inflammation or demyelination, respectively. The hematoxylin positive dots per H&E section and the demyelination area per LFB section were quantified using Image-Pro Plus 6.0 software (Media Cybernetics, Silver Spring, MD, USA).

### Anti-IL-17A antibodies therapy

Anti-IL-17A antibodies (Clone 17F3, 400 μg per mouse) were intraperitoneally injected twice weekly from Day 0 to Day 35 of MOG_35-55_ immunization to neutralize the effects of IL-17A. An IgG1 κ isotype control Ab (Clone MOPC-21) was used as control.

### Intestinal microbiota depletion

Antibiotic concoction consisting of ampicillin (10 mg ml^−1^), metronidazole (10 mg ml^−1^), vancomycin hydrochloride (5 mg ml^−1^), neomycin sulfate (10 mg ml^−1^) and amphotericin B (0.1 mg ml^−1^) was administered by gavage in a volume of 10 ml kg^−1^ body weight twice per day for 2 weeks. The clearance of intestinal flora was verified by 16s rRNA sequencing analysis.

### Fecal microbiota transplantation

For transplanting intestinal microbiota, fresh feces were collected from mice consuming individual beverages at week 9–11, and homogenized in sterile PBS under anaerobic conditions (in the above of carbon dioxide ice) at 5 mg ml^−1^; after 5 min standing, supernatants were collected and transferred to microbiota depleted mice by gavage (200 μl per mouse). Fecal microbiota gavage was administered twice per day for 3 weeks.

### 16S rRNA gene sequencing and quantitive PCR analysis

Fresh extruded stools were collected and immediately positioned in carbon dioxide ice. Feces DNA was extracted using QuickGene DNA tissue kit from Kurabo Company (Neyagawa, Japan) and sent for PCR amplification and sequencing of the V4 region of bacterial 16S rRNA genes using the Illumina MiSeq technology at BGI Co. (Shenzhen, China).

Gene catalog construction, taxonomic annotation and abundance calculation were previously described [57]. We used QIIME (Quantitative Insights into Microbial Ecology, version1.8.0) for the bio-informatical analysis. Unweighted Unifrac distance principal coordinates analysis plots were used to assess the variation between experimental groups (beta diversity). The significantly discriminative taxa between all five groups consuming individual beverages were determined by Kruskall–Wallis rank sum test with Bonferroni correction.

### Small intestine and colon LPL isolation

Small intestines and colon were dissected, Payer’s patches in small intestines were removed, and the epithelial layers were also removed by twice incubation with 1 mM DTT and 5 mM EDTA in PBS. After 20-min digestion with 1 mg ml^−1^ collagenase (type VIII, Sigma) and 300 μg ml^−1^ DNase I (Sigma) at 37 °C, total lamina propria cells were purified on 40/80% Percoll gradient.

### Intracellular cytokine and intra-nuclear Foxp3 staining

For intracellular cytokine staining, lymphocytes isolated from designated organs 18 days after immunization were stimulated, fixed and permeabilized, as previously described [58], followed by fluorescent-conjugated intracellular cytokine antibody staining. Intra-nuclear Foxp3 was stained using the Foxp3 Staining Buffer Set (eBioscience, San Diego, CA, USA).

### Luminal ATP assay

Fresh stools with similar consistency from mice were collected, weighted and gently suspended in PBS containing 0.01% NaN_3_. After centrifugation, the supernatants were collected and the levels of ATP were determined with a luciferin–luciferase assay, using an ATP assay kit (Beyotime Biotech) according to the manufacturer’s instructions.

### Statistical analysis

Data were expressed as mean±s.d., except for the clinical EAE score, which was expressed as mean±s.e.m. Differences between two groups were analyzed by a two-tailed Student’s *t* test. ANOVA was used to compare difference of data from more than two groups, and the nonparametric data (EAE scoring) were analyzed using Kruskal–Wallis test. Statistical differences were declared significant at *P*<0.05 level. Statistically significant data are indicated by asterisks (*P<0.05, **P<0.01 and ***P<0.001).

## Figures and Tables

**Table 1:** The list of ingredients of individual cola beverages

	*Coca-Cola*	*Coca-Free*	*Coca-Zero*	*Pepsi*	*Pepsi-Free*
Sucrose (W/V)	10–11%	10–11%	0	10–11%	10–11%
Caffeine	0.1 mg ml^−1^	0	0.1 mg ml^−1^	0.1 mg ml^−1^	0
Caramel	+	+	+	+	+
Phosphoric acid	+	+	+	+	+
Natural flavors	+	+	+	+	+
High fructose corn syrups	+	+	−	+	+
Aspartame	−	−	+	−	−
Sucralose	−	−	+	−	−
Acesulfame	−	−	+	−	−
Sodium benzoate	Unknown	Unknown	+	Unknown	Unknown
Sodium citrate	Unknown	Unknown	+	Unknown	Unknown
CO_2_	+	+	+	+	+
PH	2.5–4	2.5–4	2.5–4	2.5–4	2.5–4
Kcal ml^−1^	0.43	0.394	0	0.441	0.423
