# Supplementary material for: Long-term consumption of caffeine-free high sucrose cola beverages aggravates the pathogenesis of EAE in mice
Source: Cell Discov. 2017 Jun 20;3:17020–. doi: 10.1038/celldisc.2017.20 (PMC5477007; doi:10.1038/celldisc.2017.20)
Supplement: Supplementary Figures [file celldisc201720-s1.pdf]

Supplementary Figure S1. Metabolic characterization of mice consuming cola beverages. Mice were given commercial cola beverages in the dark phase (8:pm-8:am) of each day for a continuous 8 weeks. Then metabolic parameters were characterized using the comprehensive laboratory animal monitoring system (CLAMS, Columbus Instruments) for 48 h. The liquid consumption in the dark phase was measured manually. Light and dark phases are denoted by white and black rectangles on the x-axis, respectively. (**a&b**) Liquids consumption in the light and dark phase. (**c&d**) Chow intake. (**e&f**) Respiratory exchange rate (RER). (**g&h**) Spontaneous locomotor distance. (**i**) Total caloric intake (see methods for calculation). (**j**) Energy expenditure (see methods for calculation). (**k**) Body weight gain compared to original mouse weight during 8 weeks. One of the repeatable experiments is shown.

Supplementary Figure S2. Accelerated EAE disease in response to limited amounts of Coca-Free and Pepsi-Free. Mice were treated with limited amount (5ml/day/mouse) of individual cola beverages for 8 weeks and induced for EAE disease (n=7). The clinical score was monitored daily. One of the repeatable experiments is shown.

Supplementary Figure S3. Long-term consumption of HSCBs altered the community structure of microbiota and increased luminal ATP. (**a**) Mice were treated with individual cola beverages for 8 weeks and feces were used for isolation of 16s rRNA and subsequent sequencing analysis. Enriched taxa of genus in response to HSCBs are shown (n=10). (**b**) Microbiota-depleted mice were transplanted with feces from mice consuming individual cola beverages for 3 weeks. The feces were isolated from recipients and used for 16s rRNA sequencing analysis (n=7). The relative abundance of genus as in (**a**) is shown. (**c**) Three-dimensional Unweighted Unifrac PCoA (principal coordinates analysis) plots of 16s rRNA from feces donors at week 8 (n=10) and 11 (n=5). (**d**) Fresh feces with similar consistency were squeezed from mice consuming individual beverages and used for detection of luminal ATP. Data are

present as pmole/g feces. (**e**) Mice were depleted of microbiota and fecal ATP were detected and shown. (**f**) Fecal ATP from mice as in (**b**) were detected and shown. All data above are representative of two (**a-c**) or at least three (**d-f**) repeated experiments.

Supplementary Figure S4. Metabolic characterization of mice consuming high sucrose. Mice were given 10% sucrose (w/v) in the dark phase as in (Supplementary Figure S1). Then metabolic parameters were characterized for 48 h. The liquid consumption in the dark phase was measured manually. Light and dark phases are denoted by white and black rectangles on the x-axis, respectively. (**a&b**) Liquids consumption in the light and dark phase. (**c&d**) Chow intake. (**e&f**) Respiratory exchange rate (RER). (**g&h**) Spontaneous locomotor distance. (**i**) Total caloric intake. (**j**) Energy expenditure. (**k**) Body weight gain compared to original mouse weight during 8 weeks. One of the repeatable experiments is shown.

Supplementary Figure S5. Long-term consumption of high sucrose altered the community structure of microbiota and increased luminal ATP. (a) Freshly-squeezed feces with similar consistency from mice consuming 10% sucrose of H_2O were used for isolation of bacterial DNA and subsequent 16S rRNA genes sequencing analysis (n=10). The relative abundance of genus as in (Supplementary Figure S3**a**) is shown. (**b**) Fecal ATP level from mice as in (**a**) was detected and shown. (**c**) Mice were depleted of microbiota via antibiotics and fecal ATP level was detected and shown. (**d**) Feces from mice consuming high sucrose or H_2O were transferred to antibiotics-treated recipients. Fecal ATP level of recipients was detected and shown. Representative data of two (**a**) or at least three (**b-d**) individual experiments were shown.

Supplementary Table S1. Altered taxa of fecal microbiota in response to individual cola beverages and high sucrose solution.

Feces were isolated from mice consuming individual cola beverages, high sucrose or H_2O as in (Figure 3**a**, n=10) and used for isolation of fecal DNA and subsequent 16s

rRNA sequencing analysis. Altered taxa in response to individual cola beverages were summarized and shown.

Supplementary Table S2. Altered taxa of microbiota in recipients upon fecal transplantation.

Feces from mice consuming individual cola beverages, high sucrose or H2O were isolated and used for transferring into antibiotics-treated recipients as in (Figure 3**e**, n=7). Three weeks later, fecal DNA was isolated from recipients and used for 16s rRNA sequencing analysis. The altered taxa compared with H_2O feces control group were summarized and shown.

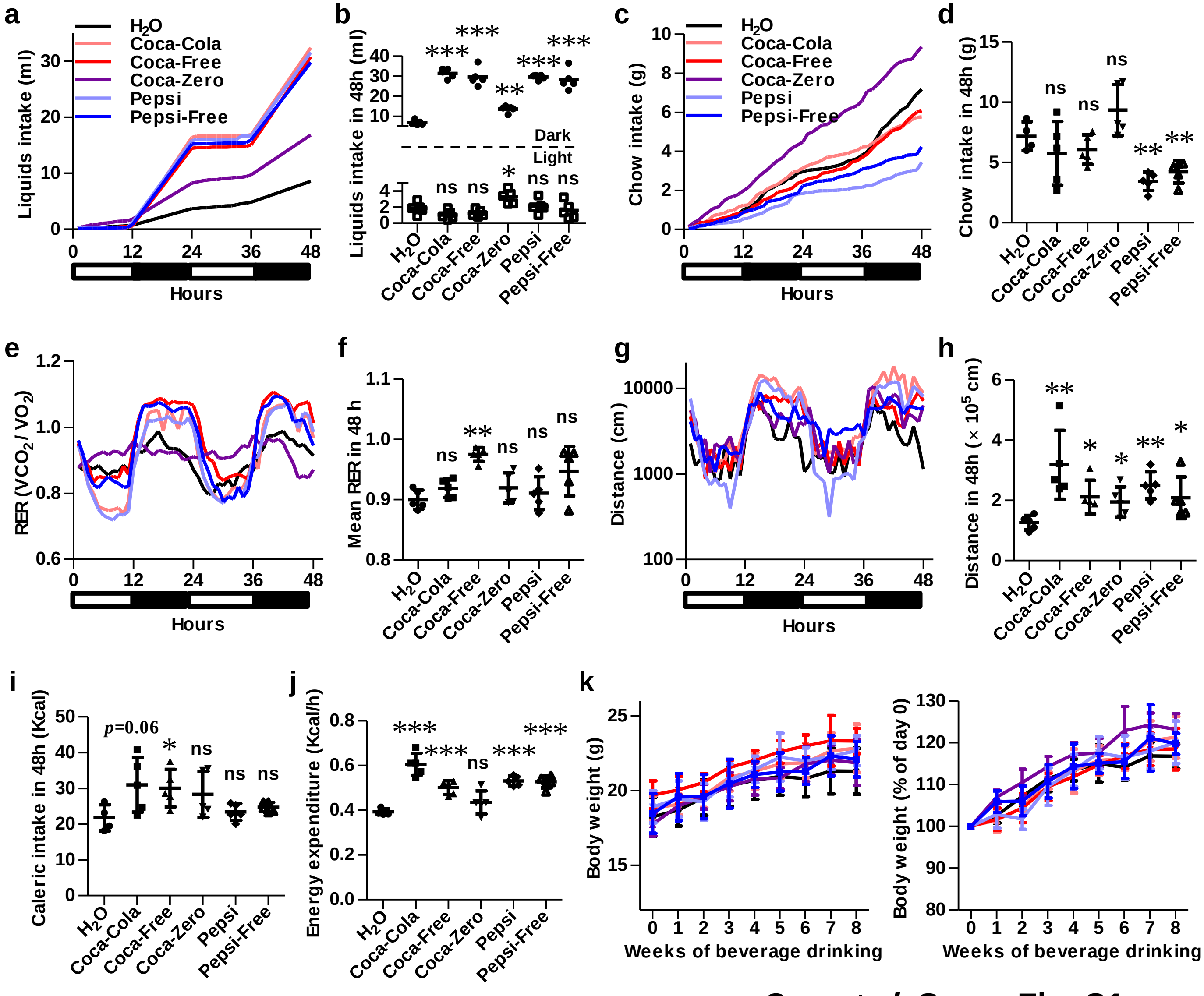

Cao *et al*. Supp. Fig. S1

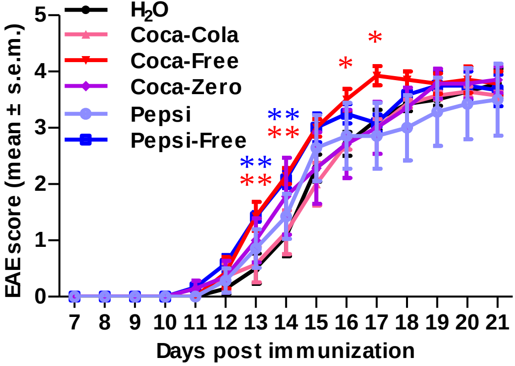

Cao *et al*. Supp. Fig. S2

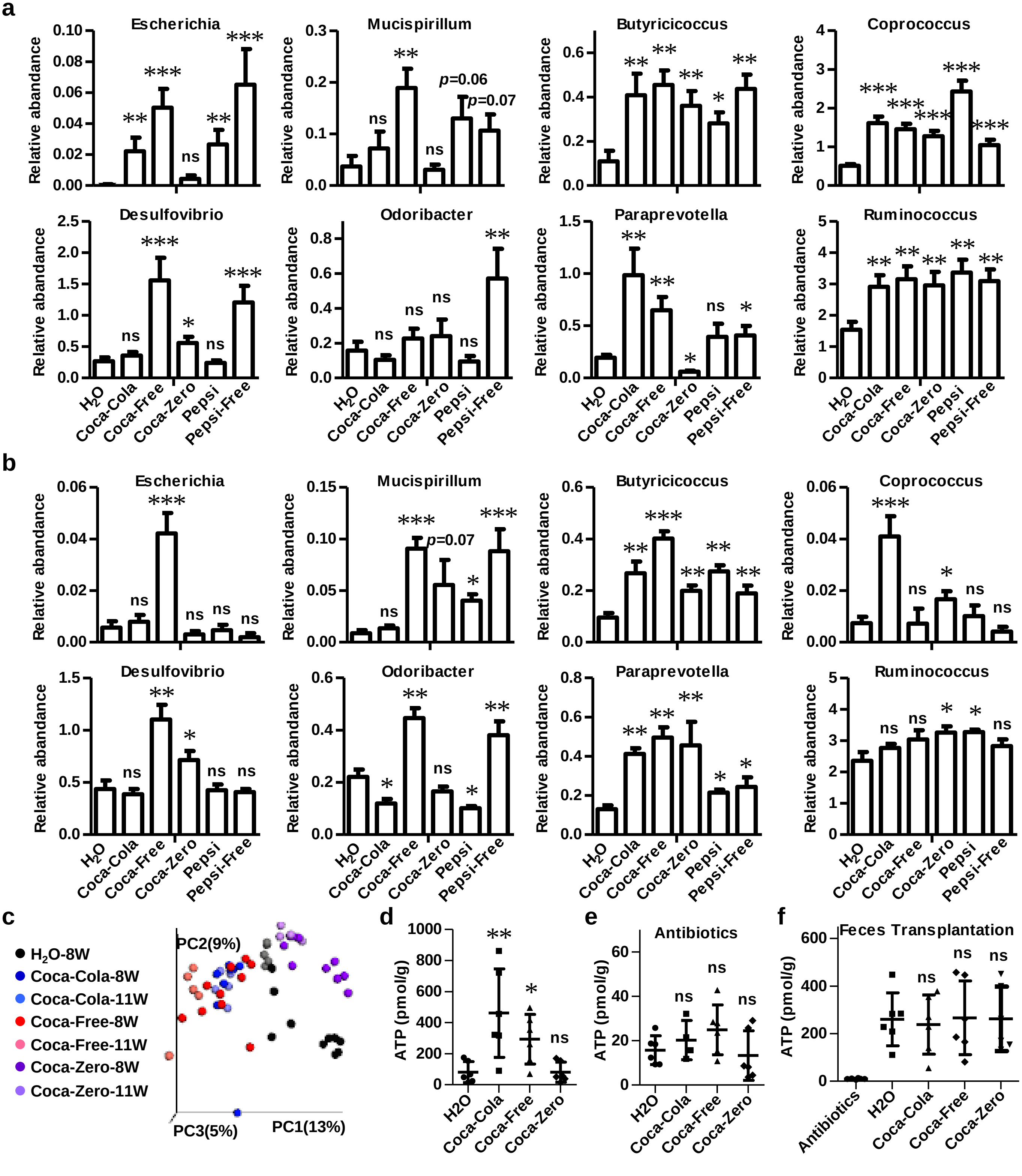

Cao *et al*. Supp. Fig. S3

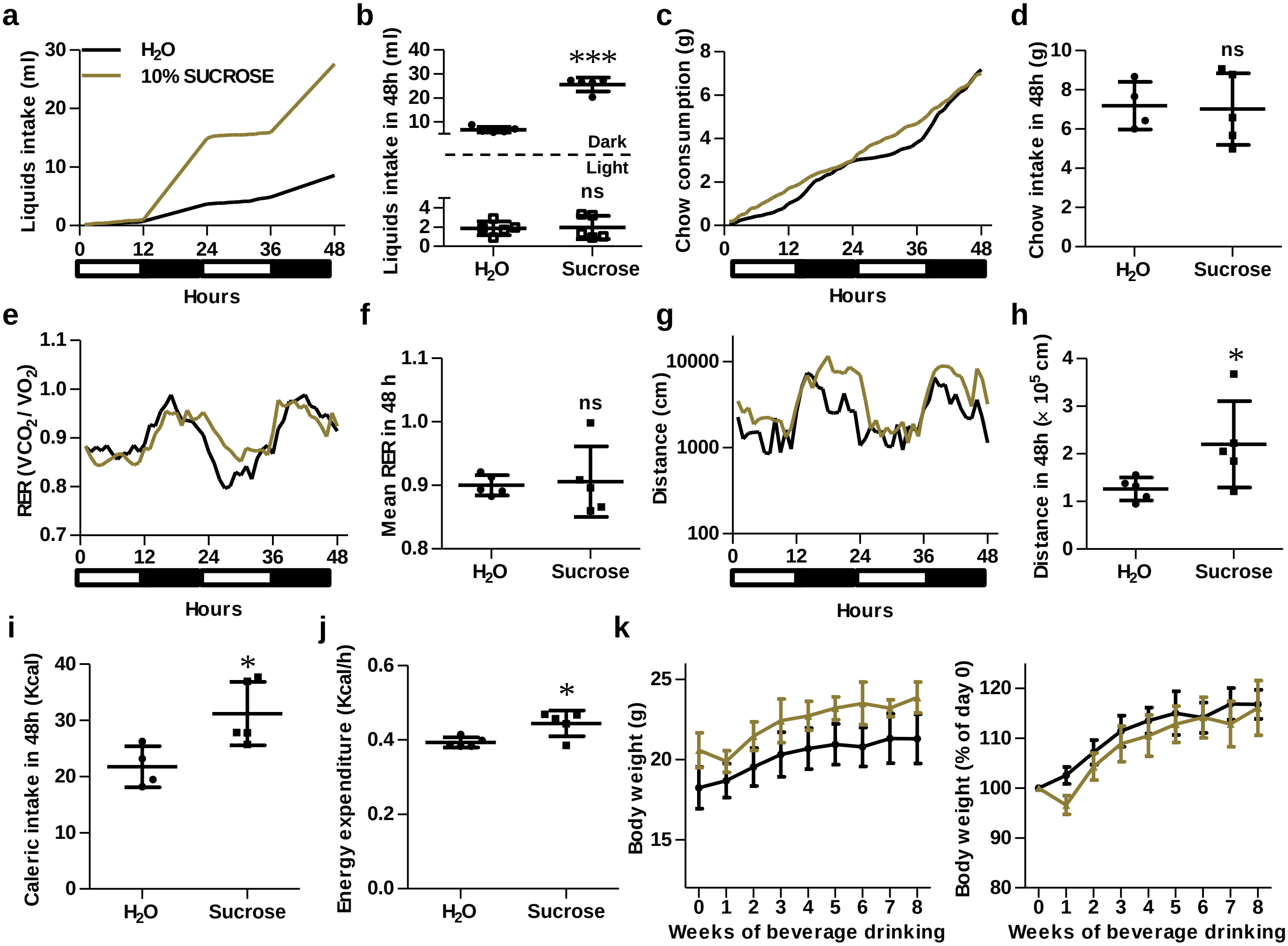

Cao *et al*. Supp. Fig. S4

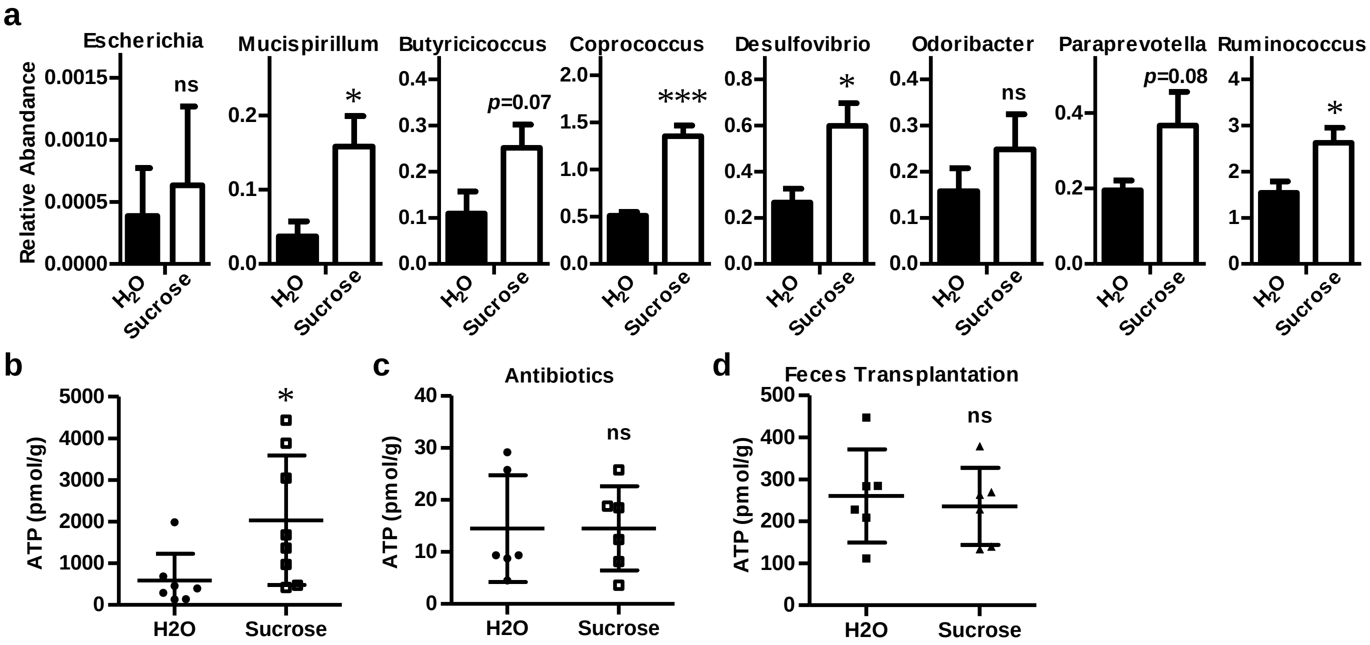

Cao *et al*. Supp. Fig. S5
